# Targeting TRPM2 in ROS-Coupled Diseases

**DOI:** 10.3390/ph9030057

**Published:** 2016-09-07

**Authors:** Shinichiro Yamamoto, Shunichi Shimizu

**Affiliations:** Division of Pharmacology, Faculty of Pharmaceutical Sciences, Teikyo Heisei University, Tokyo 164-8530, Japan; s.yamamoto@thu.ac.jp

**Keywords:** TRPM2, Ca^2+^ signaling, reactive oxygen species, ROS-coupled diseases

## Abstract

Under pathological conditions such as inflammation and ischemia-reperfusion injury large amounts of reactive oxygen species (ROS) are generated which, in return, contribute to the development and exacerbation of disease. The second member of the transient receptor potential (TRP) melastatin subfamily, TRPM2, is a Ca^2+^-permeable non-selective cation channel, activated by ROS in an ADP-ribose mediated fashion. In other words, TRPM2 functions as a transducer that converts oxidative stress into Ca^2+^ signaling. There is good evidence that TRPM2 plays an important role in ROS-coupled diseases. For example, in monocytes the influx of Ca^2+^ through TRPM2 activated by ROS contributes to the aggravation of inflammation via chemokine production. In this review, the focus is on TRPM2 as a molecular linker between ROS and Ca^2+^ signaling in ROS-coupled diseases.

## 1. Introduction

The physiological concentration of Ca^2+^ in the intracellular compartment ([Ca^2+^]_i_) is on the order of 10^−7^ M; this is markedly lower than its extracellular concentration which is in the order of 10^−3^ M [1]. Due to this difference between intracellular and extracellular Ca^2+^ concentrations, Ca^2+^ can function as a second messenger. Recently, a subset of TRP channels has attracted attention because of their permeablity to Ca^2+^. Indeed, the first *trp* gene was originally discovered in mutant fruit flies with impaired vision due to the lack of a specific Ca^2+^ influx pathway in photoreceptor cells [2]. Subsequently, a large number of TRP channel homologues were identified in vertebrates. As of today, the human TRP channel superfamily has 28 members that are divided into six subfamilies: canonical (C), vanilloid (V), melastatin (M), polycystic kidney disease (P), mucolipin (ML), and ankyrin (A), based on the homology of their protein sequences [3].

Generally speaking, the TRP protein has six putative transmembrane domains and a pore region between the fifth and sixth transmembrane domains. TRP proteins assemble into homo- or heterotetramers in order to form functional channels [4,5]. The TRPC subfamily shows the greatest homology to the *Drosophila* TRP protein. TRPC channels are downstream targets to phospholipase C activation following receptor stimulation [6,7,8,9].

The TRPV subfamily (TRPV1 to V6) was named after its founding member, the vanilloid (capsaicin) receptor TRPV1. TRPV channels are polymodal and their activators range from physical and chemical stimuli including heat (TRPV1, TRPV2, TRPV3, and TRPV4) [10,11,12,13,14,15,16], through protons (TRPV1) [17] and osmotic stress (TRPV4) [18,19], to capsaicin, the pungent principle in hot peppers (TRPV1) [10]. The TRPM subfamily has eight members. Its best known member is the cold-responsive menthol receptor, TRPM8 [20,21]. The TRPP subfamily includes TRPP1 and TRPP2, which are encoded by the *PKD1* and *PKD2* genes, respectively. *PKD1* and *PKD2* are the genes responsible for autosomal dominant polycystic kidney disease. TRPP1 is thought to interact with TRPP2, which functions as a receptor for mechanical stimuli such as shear stress [22,23].

The TRPML subfamily is composed of TRPML1 and its homologues. A mutation in the *MCOLN1* gene encoding TRPML1 causes mucolipidosis type IV. TRPML1 localizes in lysosomes and late endosomes and is activated by phosphoinositol (3,5)-bisphosphate [24,25].

TRPA1 (named after the large N-terminal domain with 17 predicted ankyrin repeats) is the sole member of the TRPA subfamily [26]. It is activated by irritant compounds such as exhaust fumes and allyl isothiocyanate in mustard oil. The cold activation of TRPA1 remains controversial [27,28]. Interestingly, TRPA1 is activated by both hyper- and hypoxia via oxidative modification of its cysteine residues and the dehydroxylation of the proline residues [29].

Traditionally, reactive oxygen species (ROS) are regarded as non-specific toxins that cause cell and tissue damage [30]. However, recently ROS have been identified as signal-transduction molecules [31]. For example, the oxidative stress-sensitive transcriptional factor Keap1, and the signal-transduction molecule ASK1, are activated by ROS to mediate a number of cellular responses [32,33]. The second member of the TRP melastatin subfamily, TRPM2, is a Ca^2+^-permeable non-selective cation channel. TRPM2 is expressed broadly in neuronal cells, myocytes, pancreatic β cells, and immune cells such as T lymphocytes, monocytes/macrophages, and neutrophils [34,35,36,37,38,39,40,41,42]. TRPM2 is activated by oxidative stress including H_2_O_2_. In other words, TRPM2 functions as a sensor for oxidative stress. Indeed, TRPM2 is more sensitive to ROS than other TRPs including TRPC5, TRPV1 and TRPA1 (which is activated by ROS via oxidative modifications to its cysteine residues).

Large amounts of ROS are generated under pathological conditions that, in turn, contribute to the development and maintenance of various disease states [43]. TRPM2 converts ROS-induced oxidative stress into Ca^2+^ signaling; this Ca^2+^ signaling has been implicated in the aggravation of a number of diseases. In this review, the focus is on TRPM2 as a molecular linker between ROS and Ca^2+^ signaling.

## 2. TRPM2 Activators and Inhibitors

Among TRP channels TRPM2 is unique in that it contains a NudT9-Homology (NUDT9-H) domain at its cytosolic C-terminal region. Although NUDT9-H shares some homology with NUDT9 ADP-ribose hydrolase, its ADP-ribose hydrolase activity is low. In addition to the full-length TRPM2, several truncated splice variants have been described, including: (1) TRPM2-ΔN (containing a deletion of amino acids 538–557 in the N-terminus); (2) TRPM2-ΔC (deletion of amino acids 1292–1325 in the C-terminus), and (3) TRPM2-S (S for short) that lacks the four C-terminal transmembrane domains, putative Ca^2+^-permeable pore region, and the entire C terminus [39,41,44].

The activation of TRPM2 is triggered by the binding of ADP-ribose to the NUDT9-H domain [45]. Since the NUDT9-H domain of TRPM2-ΔC is partially missing, TRPM2-ΔC is not activated by ADP-ribose. Nicotinic acid adenine dinucleotide phosphate (NAADP), cADP-ribose, and Ca^2+^ exert synergistic effects on ADP-ribose-induced TRPM2 activation. Moreover, these agents are also capable of activating TRPM2 by themselves [36,46,47,48,49,50].

In neutrophils, resting ADP-ribose levels approach 5 μM [50] which is sufficient to induce the activation of TRPM2 by increasing [Ca^2+^]_i_. The IQ-like motif in the calmodulin-binding domain at the N-terminal region, rather than the NUDT9-H domain, is thought to play a pivotal role in the Ca^2+^-induced activation of TRPM2 [49]. Using inside-out patch recordings, Csanády and colleagues have investigated the direct activation of TRPM2; they found that neither cADP-ribose nor NAADP is able to directly activate TRPM2 [51]. On the other hand, they identified ADP-ribose-2’-phosphate as a direct TRPM2 agonist [52].

Silent information regulator-2 (SIR2), a member of the sirtuin family, is a nicotinamide adenine dinucleotide (NAD^+^)-dependent protein deacetylase. SIR2 removes acetyl groups from acetylated substrates, and transfers them to NAD^+^. Nicotinamide and O-acetylated-ADP-ribose (OAADPr) are produced as a result of this reaction. OAADPr was reported to activate TRPM2 by binding to the NUDT9-H domain. This implicates SIR2 in TRPM2 regulation [53].

NAD^+^ was also reported to directly gate TRPM2, although a high concentration (in the mM range) of NAD^+^ is required for this response [54]. However, purified NAD^+^ fails to activate TRPM2 [52]. This apparent contradiction was explained by the presence of the NAD^+^-degradation product ADP-ribose, a known TRPM2 agonist, in the non-purified NAD^+^ [46,55]. CD38 is an ectoenzyme that catalyzes the production of cADP-ribose and ADP-ribose from its substrate, NAD^+^ [56]. CD38 is implicated in the activation of TRPM2 via production of cADP-ribose and/or ADP-ribose [38,40,57].

Arguably the most important activator of TRPM2 is oxidative stress induced by ROS, including H_2_O_2_ [54]. It has been postulated that the activation of TRPM2 by oxidative stress is triggered via ADP-ribose production. Mitochondria are a major source of ADP-ribose. In mitochondria, ADP-ribose is generated by the oxidative stress-induced hydrolysis of NAD^+^ [55]. In the nucleus, poly(ADP-ribose) polymerase-1 (PARP-1) plays an important role in repairing DNA damage in response to oxidative stress. The binding of PARP-1 to impaired DNA hydrolyzes NAD^+^, leading to the production of nicotinamide and ADP-ribose. In turn, ADP-ribose is built into various nuclear proteins, resulting in the activation of DNA repair and stimulation of nuclear factor-mediated transcription [58,59,60,61]. Free ADP-ribose is generated following the degradation of poly(ADP-ribose) by poly(ADP-ribose) glycohydrolase (PARG) [61].

Pharmacological [62] or genetic manipulation of PARP-1 [63] blocks H_2_O_2_-induced TRPM2 activation. Conversely, H_2_O_2_-induced TRPM2 activation is enhanced at body temperature by hydroxyl radical production [64,65]. The hydroxyl radical produced by the reaction of H_2_O_2_ with intracellular Fe^2+^ (Fenton reaction) stimulates the PARP-1/PARG pathway, which leads to the activation of TRPM2. The phosphorylation of tyrosine residues in TRPM2 is thought to represent an important mechanism underlying the activation of TRPM2 by H_2_O_2_. The phosphorylation/dephosphorylation state is regulated by protein tyrosine phosphatase-L1 [66].

The short splice variant of TRPM2, TRPM2-S, was shown to interact with the full-length TRPM2. The TRPM2-S/full-length TRPM2 complex is not activated by H_2_O_2_ [39]. Therefore, TRPM2-S may function as a dominant negative modulator of TRPM2.

A large number of TRPM2 blockers have been reported. Adenosine monophosphate [46] and 8-bromo-ADP-ribose [38] inhibit ADP-ribose-induced TRPM2 activation by preventing the binding of ADP-ribose to the NUDT9-H domain. The antifungal agents clotrimazole and econazole [67], the antipyretic agent flufenamic acid [68], 2-aminoethoxydiphenyl borate (2-APB) [69], *N*-(p-amylcinnamoyl) anthranilic acid (ACA) [70], and curcumin, the active principle in turmeric [71], are TRPM2 channel blockers. PARP inhibitors (e.g., SB750139-B, PJ34, and DPQ) were also reported to prevent the activation of TRPM2 in response to oxidative stress. However, these inhibitors have no effect on ADP-ribose-induced TRPM2 activation.

Iron chelators were shown to attenuate H_2_O_2_-induced TRPM2 activation [64]. Surprisingly, the JAK2 inhibitor AG490 was also found to prevent TRPM2 activation by H_2_O_2_ [72]. It was, however, suggested that AG490 ameliorates H_2_O_2_-induced TRPM2 activation by scavenging hydroxyl radicals rather than inhibiting of JAK2. The AG490-related compounds, AG555 and AG556, exert an even stronger inhibitory effect on H_2_O_2_-induced TRPM2 activation than AG490 [73].

## 3. ROS Production under Pathological Conditions

### 3.1. Inflammation

Inflammation is a complex biological reaction to injury and/or infection. During inflammation, immune cells are transported from the blood stream into the damaged tissue in an attempt to eliminate the harmful agents and to initiate the process of healing and repair. However, when inflammation becomes chronic, it may exacerbate tissue damage and pose severe health risks.

At the inflamed sites, phagocytes (e.g., macrophages and neutrophils) digest the harmful agents which play an important role in their removal. During phagocytosis, oxygen consumption in the phagocytes is increased. This phenomenon is known as the “respiratory burst”: oxygen is utilized for superoxide anion (^·^O_2_^−^) production by NADPH oxidase [74]. During bacterial phagocytosis, bacteria are engulfed by the plasma membrane, leading to the formation of phagosomes. Then NADPH oxidase activated and the resultant ^·^O_2_^−^ contributes to bacterial killing. NADPH oxidase (NOX) is composed of several isoforms. Seven isoforms, termed as NOX1–5 and DUOX1–2, have been identified as catalytic subunits. These isoforms are localized in the plasma membrane and catalyze electron transport from the electron donor NADPH to oxygen, leading to the production of ^·^O_2_^−^. In phagocytes, NOX2 is strongly expressed and interacts with the membrane protein p22^phox^ [75]. The small G-protein RAC, and the cytosolic proteins p40^phox^, p47^phox^, and p67^phox^ are also known to activate NOX2.

**Figure 1:**
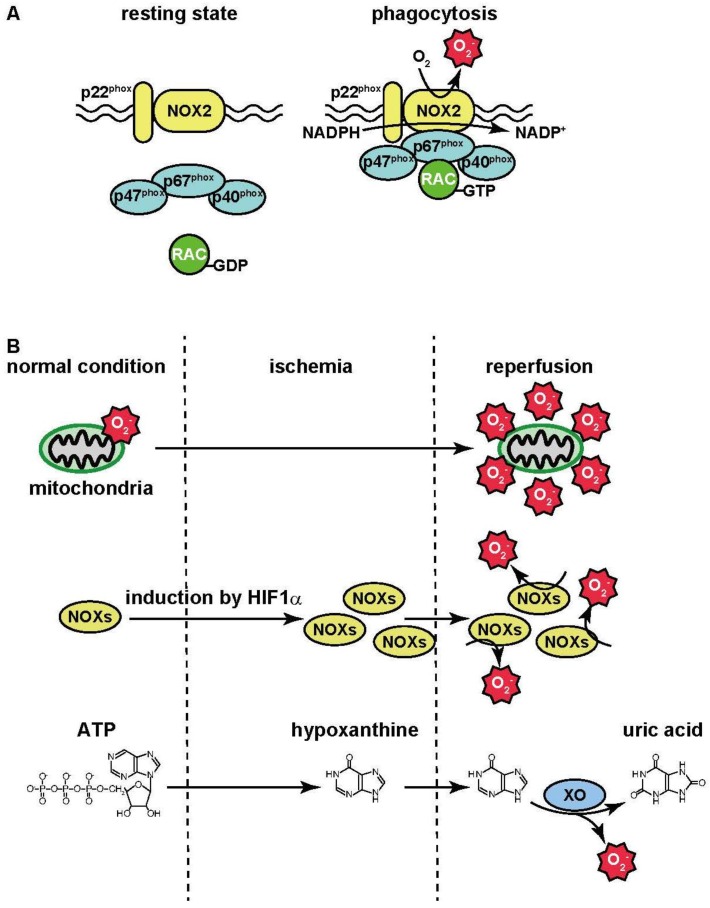
ROS production during inflammation and ischemia-reperfusion. (**A**) In resting state, cytosolic activators such as p40^phox^, p47^phox^, p67^phox^ and small G protein RAC do not interact with NOX2-p22^phox^ complex. These activators translocate to the plasma membrane during phagocytosis and interact with the NOX2-p22^phox^ complex. Electrons derived from NADPH are transferred through the complex to molecular oxygen, leading to ^·^O_2_^−^ production; (**B**) Oxidative phosphorylation is initiated by electron transport from NADH and/or FADH_2_ to the electron transport chain in the mitochondrial inner membrane. The electron transport chain is composed of complexes I–IV. Electrons derived from NADH and FADH_2_ are fed to complex I and complex II, respectively. They are then transferred to complexes in ascending order of the redox potential, which release free energy. Molecular oxygen accepts electrons for the formation of H_2_O. On the other hand, the electron transport chain uses free energy derived from electron transport to pump H^+^ out of the matrix, thereby creating proton gradient across the mitochondrial inner membrane. By utilizing energy released by the influx of H^+^ into the matrix, ADP is phosphorylated, resulting in the generation of ATP. ^·^O_2_^−^ is generated by the leakage of electrons from complexes I and III in the electron transport chain. The activity of the electron transport chain generates a relatively small amount of ^·^O_2_^−^ under normal conditions, but its production may be greatly magnified by events occurring during ischemia-reperfusion. The expression of NOX isoforms is up-regulated by HIF1α during ischemia, and then NADPH oxidase then generates large amounts of ROS by reoxygenation during reperfusion. During ischemia, ATP is catabolized into hypoxanthine.

The production of ^·^O_2_^−^ by NOX2 does not occur in a resting state. During phagocytosis, these activators translocate to the plasma membrane and interact with the NOX2/p22^phox^ complex; this, in turn, triggers ^·^O_2_^−^ production following the activation of NOX2 (Figure 1A). In addition, ^·^O_2_^−^ is converted to H_2_O_2_ by the superoxide dismutase. These ROS contribute to killing bacteria.

Lipopolysaccharide (LPS), found in the outer membrane of Gram-negative bacteria, is a prototypical trigger of sepsis that elicits a strong immune response in animals. The LPS receptor is toll-like receptor-4 (TLR4) which associates with several adaptor molecules such as MyD88 [76]. The activation of TLR4 in response to LPS triggers immune responses including the production of cytokines and ROS accompanied by the activation of NADPH oxidase [77]. Cytokines (e.g., tumor necrosis factor, TNF) are released from immune cells and accumulate at the sites of inflammation. These molecules act in concert to organize the inflammatory network and produce large amount of ROS. The source of ROS appears to be the mitochondria rather than the NADPH oxidase [77,78,79].

### 3.2. Ischemia-Reperfusion

Ischemia-reperfusion injury is caused by re-oxygenation during reperfusion following the lack of oxygen during ischemia. Ischemia-reperfusion generates harmful substances that aggravate the tissue injury. This is a major mechanism of tissue damage during stroke and myocardial infarction. The hydroxyl radical scavenger, edaravone, is used as a neuroprotective agent in the management of patients with ischemic brain injury and amyotrophic lateral sclerosis (Lou Gehrig’s Disease). During ischemia-reperfusion injury, mitochondria are the major source of ROS. Electrons leaked from the mitochondrial electron transport chain are transferred to molecular oxygen, resulting in the production of ^·^O_2_^−^. The activity of the electron transport chain generates a relatively small amount of ^·^O_2_^−^ under normal conditions, but its production may be greatly magnified by events occurring during ischemia-reperfusion (Figure 1B) [80].

NADPH oxidase also contributes to ROS production during ischemia-reperfusion. NOXs are present in blood vessels [81] where their expression is regulated by the hypoxia-sensitive transcriptional factor, hypoxia-inducible factor-1α (HIF1α) [81]. The expression of NOX isoforms is thus up-regulated by the lack of oxygen during ischemia. Then NOX generates large amounts of ROS during reperfusion (Figure 1B).

During ischemia, a failure in the generation of ATP also occurs concurrently with ATP consumption, leading to the depletion of ATP. ATP is eventually catabolized into hypoxanthine. Xanthine oxidase catalyzes two steps including the formation of xanthine from hypoxanthine, and the formation of uric acid from xanthine. Electrons are also generated in this process and transferred to molecular oxygen, leading to the formation of ^·^O_2_^−^ [82] (Figure 1B). In summary, several factors contribute to ROS production during ischemia-reperfusion.

## 4. ROS-Coupled Diseases and TRPM2

### 4.1. Inflammatory Diseases

#### 4.1.1. TRPM2-Mediated Chemokine Production

TRPM2 contributes to the aggravation of inflammation [40]. In monocytes/macrophages, Ca^2+^ influx through TRPM2 activated by ROS stimulates the production of the chemokine, CXCL2. CXC chemokines, such as macrophage inflammatory protein-2 (CXCL2), exhibit potent neutrophil chemotactic activity [83].

Dextran sulfate sodium (DSS)-induced colitis is a mouse model of human ulcerative colitis. In the colon of DSS-treated wild-type (WT) mice, the expression of CXCL2 was markedly increased in monocytes/macrophages. By contrast, CXCL2 expression was strongly suppressed in the colon of *Trpm2* KO mice following DSS challenge. The number of recruited neutrophils was also significantly reduced in the colon of DSS-treated *Trpm2* KO mice, presumably as a consequence of reduced CXCL2 levels, but their function was intact. No difference was noted in the number of macrophages in the inflamed colon of WT and *Trpm2* KO mice. The bone marrow output of neutrophils was normal, as was their accumulation into the abdominal cavity after intraperitoneal injection of chemokines. Last, DSS-treated *Trpm2* KO mice did not exhibit weight loss and/or ulceration of the colon, suggesting that *Trpm2* KO mice were largely protected from DSS-mediated colitis. Combined, these findings imply that TRPM2-mediated chemokine production in monocytes/macrophages is an important mechanism underlying the progression of DSS-induced ulcerative colitis.

TRPM2-dependent CXCL2 production was also implicated in the carrageenan-induced inflammatory pain and sciatic nerve ligation models [84]. The carrageenan-induced pain model is a widely used and reliable model for inflammatory pain. Sciatic nerve ligation causes neuropathic pain. Both CXCL2 production and neutrophil infiltration were attenuated in *Trpm2* KO mice. By contrast, the recruitment of F4/80-positive macrophages was not altered in the inflamed paw or around the injured sciatic nerve. Importantly, both mechanical allodynia and thermal hyperalgesia were attenuated in *Trpm2* KO mice. Based on these observations one may argue that TRPM2 expressed in macrophages aggravates pronociceptive inflammatory responses to induce inflammatory and neuropathic pain through neuroinflammation-mediated sensitization of the pain-signaling pathway.

TRPM2 in alveolar epithelial cells plays an important role in bleomycin-induced lung injury [85]. Bleomycin is a glycopeptide antibiotic with potent antitumor activity. It is used in the management of squamous cell carcinoma, testicular cancers, and lymphomas. The antitumor activity of bleomycin was attributed to its ability to cause DNA damage in the cancer cells through the production of oxygen radicals. A major dose-limiting side-effect of bleomycin is lung injury. In mice treated with bleomycin, the secretion of CXCL2 from alveolar epithelial cells was attenuated in *Trpm2* KO compared to WT. It was unexpected because alveolar macrophages (which have higher expression of TRPM2 than alveolar epithelial cells) were believed to be the main source of CXCL2 in response to bleomycin. The secretion of CXCL2 from alveolar epithelial cells was essential for neutrophil recruitment and the secretion of inflammatory cytokines including tumor necrosis factor α and interleukin-1β. Taken together, these findings imply that TRPM2-mediated CXCL2 production in alveolar epithelial cells is responsible for the aggravation of bleomycin-induced lung damage.

#### 4.1.2. LPS-Induced Inflammatory Responses and TRPM2

The contribution of TRPM2 to LPS-induced lung inflammation is poorly understood. In vitro, activation by LPS of TRPM2 in monocytes and cultured microglia is involved in the generation of inflammatory cytokines [40,84,86]. By contrast, in vivo studies found no difference between WT and *Trpm2* KO mice in the secretion of inflammatory cytokines and the infiltration of inflammatory cells into the lungs following LPS administration [85,87]. Therefore, other signaling pathways (e.g., TLR4-mediated signaling) rather than Ca^2+^ signaling via TRPM2 may play a pivotal role in LPS-induced lung inflammation in vivo.

Adding to the confusion, recently Di et al suggested a protective anti-inflammatory role for TRPM2 during LPS-induced lung inflammation [88]. In LPS-treated *Trpm2* KO mice lung injury (including cytokine production and the infiltration of inflammatory cells into the lungs) was exacerbated compared to WT animals. In their experimental model, following LPS administration Ca^2+^ influx via TRPM2 was triggered in phagocytes such as neutrophils. The influx of Ca^2+^ depolarized the plasma membrane, contributing to the inhibition of NADPH oxidase. This protective mechanism was absent in the *Trpm2* KO animals. Therefore, *Trpm2* KO phagocytes overproduced ROS, resulting in the exacerbation of LPS-induced lung injury.

#### 4.1.3. Functional Roles of TRPM2 during Infection

TRPM2 may play an important protective role during bacterial infections. For example, the Gram-negative bacterium *Francisella tularensis* (the agent responsible for tularemia) is equipped with an antioxidant system to escape the host immune response. Although *Francisella* is phagocytized by macrophages, it protects itself from ROS-mediated killing by inhibiting the formation of the NADPH oxidase complex [89]. Catalase (that converts H_2_O_2_ into H_2_O and oxygen) also belongs to the antioxidant systems in *Francisella*.

By using a catalase-deficient *F. tularensis* strain, Shakerley et al. suggested that TRPM2 may play a central role in macrophages during bacterial infection [90]. Although macrophages infected with *F. tularensis* showed marginal TRPM2 activation, the influx of Ca^2+^ through TRPM2 was sufficient to induce immune responses such as interleukin-6 (IL-6) production in macrophages infected with catalase-deficient *F. tularensis*. During *Listeria monocytogenes* infection, TRPM2 was found to contribute to innate immunity [91]. In *Trpm2* KO mice infected by *L. monocytogenes*, the production of IL-12 and interferon-γ was diminished. Consequently, *Trpm2* KO mice were more susceptible to *L. monocytogenes* infection.

Formyl-methionyl-leucyl-phenylalanine (fMLP) is a secreted bacterial product that serves as a neutrophil chemotactic factor. fMLP activates CD38 by binding to its receptor. *Cd38*-deficient mice display disturbed Ca^2+^ signaling and neutrophil chemotaxis in response to fMLP [92]. As described above, CD38 is an ectoenzyme that catalyzes the production of cADP-ribose and ADP-ribose from its substrate, NAD^+^. fMLP-induced Ca^2+^ influx and neutrophil chemotaxis were significantly suppressed in the *Trpm2*-deficient neutrophils, suggesting that TRPM2 is a molecular entity that links ADP-ribose produced by CD38 to Ca^2+^ signaling [38,40].

#### 4.1.4. NLRP3 Inflammasome and TRPM2

The NOD-like receptor family pyrin domain containing-3 (NLRP3) “inflammasome” is composed of NLRP3, apoptosis-associated speck-like protein (ASC), and caspase-1. NLRP3 associates with the adaptor protein ASC in response to danger-associated stimuli. In order to form an active inflammasome complex, the NLRP3-ASC complex needs to bind caspase-1. This interaction results in the caspase-1-dependent processing of cytoplasmic targets, including the pro-inflammatory cytokines IL-1β and IL-18. Mature cytokines are then released from the cells [93]. The influx of Ca^2+^ via TRPM2 activated by ROS was suggested to participate in the activation of the NLRP3 inflammasome [94].

Particulate substances (e.g., liposomes and urate crystals) induce the production of ROS, partially mediated by the leakage of electrons from the mitochondrial electron transport chain [94,95]. These particulates also initiate a ROS-dependent Ca^2+^ influx via TRPM2; this, in turn, contributes to the secretion of IL-1β, accompanied by NLRP3 inflammasome activation. In *Trpm2*-disrupted macrophages, impaired NLRP3 inflammasome activation and interleukin-1β secretion was observed. Furthermore, *Trpm2* KO mice are resistant to particulate-induced and IL-1β-mediated peritonitis [94].

### 4.2. Ischemia-Reperfusion Injury

#### 4.2.1. Brain

Ca^2+^ signaling influences a wide array of biological responses, including gene expression, neuronal growth, neurotransmitter release, and, ultimately, cell death. In other words, Ca^2+^ can exert both protective and deleterious effects on neuronal cells [96,97]. TRPM2 is believed to be responsible for the H_2_O_2_-induced Ca^2+^ influx that mediates cell death in various tissues [54] including rat cortical neurons [34]. There is good evidence that the influx of Ca^2+^ via TRPM2 contributes to neuronal cell death during ischemia-reperfusion injury both in vitro (oxygen and glucose deprivation, OGD, followed by re-oxygenation) and in vivo (brain ischemia-reperfusion induced by transient middle cerebral artery occlusion, tMCAO). Interestingly, there appears to be a sex-related difference in cell death [98,99,100]. When both male and female WT and *Trpm2* KO mice were subjected to tMCAO, male *Trpm2* KO mice had smaller infarct volumes than matched WT mice. By contrast, *Trpm2* KO had no protective effect on infarct volumes in female mice. In a second set of experiments, clotrimazole was used as a TRPM2 inhibitor. Clotrimazole reduced infarct volumes in male WT mice subjected to tMCAO. This beneficial effect was absent in *Trpm2* KO mice. Clotrimazole had no effects either on infarct volumes in castrated male mice. However, androgen replacement restored clotrimazole protection in castrated mice. Taken together, these findings suggest that androgen signaling contributes to TRPM2-dependent brain injury during ischemia-reperfusion. One may argue that androgen signaling stimulates PARP-1 which is necessary for the engagement of TRPM2 in ischemic injury in the male brain. However, other mechanisms may also exist because cell death was induced by OGD in neurons isolated from male embryos and cultured in sex steroid-free medium.

There is preliminary evidence that the *N*-methyl-d-aspartate glutamate receptor (NMDA-R) subunit expression pattern is altered in *Trpm2* KO mice [101]. NMDA-R is a heteromer composed of the obligatory GluN1 subunit along with other GluN subunits including GluN2A and GluN2B. An increase in the activity of GluN2A-containing NMDA-R is known to increase the phosphorylation of Extracellular Signal Regulated Kinase-1 (ERK) and AKT, thereby promoting pro-survival mechanisms in the cell. In contrast, an increase in the activity of GluN2B-containing NMDA-R inhibits pro-survival mechanisms [101]. *Trpm2* KO mice subjected to tMCAO showed smaller infarcts than WT mice, and OGD-induced cell death was reduced in hippocampal neurons prepared from *Trpm2* KO embryos. The expression of GluN2B and GluN2A was reduced and increased, respectively, in the hippocampus by the disruption of *Trpm2*. The ERK/AKT pathway was activated in the hippocampus of *Trpm2* KO mice.

As described above, stimulation of the NMDA-R (that contains GluN2A) activates the ERK/AKT pathway that, in turn, promotes pro-survival mechanisms. In the OGD model, the application of known GluN2A antagonists eliminated the neuroprotection in the hippocampal neurons isolated from *Trpm2* KO mouse embryos. This implies that increases in GluN2A by the disruption of *Trpm2* protect neurons from ischemia-reperfusion-induced cell death.

Migration of immune cells including neutrophils from the blood stream into the brain also plays an important role in ischemia-reperfusion brain injury [102]. As mentioned above, the size of the infarct induced by tMCAO was significantly smaller in *Trpm2* KO mice than in WT mice. WT mice transplanted with bone marrow obtained from *Trpm2* KO animals showed significantly smaller brain infract in the tMCAO model than *Trpm2* KO animals reconstituted with bone marrow from WT mice. This experiment supports the pivotal role of TRPM2 expressed in bone marrow-derived immune cells in the pathomechanism of ischemia-reperfusion brain injury.

#### 4.2.2. Heart

Conflicting results have been reported regarding the function of TRPM2 in heart injury during ischemia-reperfusion. Cheung and colleagues reported that TRPM2 protected the heart against ischemia-reperfusion injury [35,103,104]. TRPM2 was expressed in the sarcolemma and transverse tubules of adult cardiomyocytes. After reperfusion following coronary artery occlusion, no significant differences were observed in infarct sizes between WT and *Trpm2* KO mice. The heart function was, however, compromised in *Trpm2* KO mice. ROS levels in left ventricular myocytes were significantly higher in *Trpm2* KO mice than in WT mice after ischemia-reperfusion. The levels of superoxide dismutase and its transcriptional factors, forkhead box transcription factor and HIF, were lower, whereas that of the NADPH oxidase catalytic subunit, NOX4, was higher in *Trpm2* KO mouse hearts subjected to ischemia-reperfusion [35]. In addition, mitochondrial proteins and complex I subunits were down-regulated in *Trpm2* KO mouse heart [103]. These alterations in protein expression triggered by ROS overproduction and mitochondrial dysfunction in *Trpm2* KO mouse heart may be responsible for the heart dysfunction.

Another study, by contrast, found that heart functions were improved in *Trpm2* KO mice, suggesting that a deficiency in TRPM2 protects heart against ischemia-reperfusion injury [105]. Albeit TRPM2 mRNA expression was observed in the heart, its level was markedly lower than that in neutrophils or neurons. Neutrophilic infiltration of the heart after ischemia-reperfusion was reduced in *Trpm2* KO mice. It was speculated that TRPM2 expressed in neutrophils, rather than the heart, is important for ischemia-reperfusion heart injury. Indeed, in isolated hearts infarct sizes were significantly smaller in the heart obtained from *Trpm2* KO mice and perfused with *Trpm2* KO neutrophils compared to *Trpm2* KO hearts perfused with WT neutrophils. Likewise, infarct sizes were significantly larger in the heart of *Trpm2* KO mice carrying WT neutrophils compared to the heart of *Trpm2* KO mice with TRPM2-deficient neutrophils, suggesting that the activation of neutrophil TRPM2 during reperfusion has an important role in the development of myocardial infarction.

By using a cardiac-specific *Trpm2* KO mice, Cheung and colleagues recently reported a functional role for TRPM2 in the heart [104]. Similar to their studies using conventional *Trpm2* KO mice, heart functions after ischemia-reperfusion were aggravated in the cardiac-specific *Trpm2* KO mice. On the other hand, significant differences in infarct sizes were not observed between the WT and cardiac-specific *Trpm2* KO animals. In summary, the role of TRPM2 in cardiac ischemia-reperfusion injury remains controversial.

#### 4.2.3. Kidneys

In the kidneys, TRPM2 is thought to contribute to the aggravation of renal injury and ROS production after ischemia-reperfusion [106]. TRPM2 is expressed in the proximal tubules. The disruption of *Trpm2* protects kidneys against ischemia-reperfusion injury. This involvement of TRPM2 was shown to be linked to the presence of TRPM2 in parenchymal cells rather than hematopoietic cells. Oxidative stress accompanied by the activation of NADPH oxidase was triggered in WT, but in *Trpm2* KO, mouse kidneys subjected to ischemia-reperfusion. Ca^2+^ influx via TRPM2 participated in the activation of RAS-related C3 botulinum toxin substrate-1 (RAC1), an essential factor for the activation of NADPH oxidase.

### 4.3. Other Diseases and Injuries

#### 4.3.1. Acetaminophen-Induced Liver Injury

Acetaminophen is an antipyretic analgesic drug. Acetaminophen overdose (accidental or intentional) is a well-known cause of potentially fatal liver injury [107,108]. Acetaminophen is mainly metabolized into a non-toxic compound via glucuronidation and a sulfation reaction. On the other hand, a small amount of acetaminophen is converted to the toxic compound, *N*-acetyl-parabenzo-quinoneimine (NAPQI). NAPQI is then metabolized into a non-toxic compound via glutathione conjugation. NAPQI is responsible for acetaminophen-induced liver injury. NAPQI was shown to deplete intracellular glutathione levels, leading to the production of ROS [109]. TRPM2 has been implicated in acetaminophen-induced liver injury [109]. H_2_O_2_ and acetaminophen induce Ca^2+^ influx into hepatocytes in a TRPM2-dependent manner. Acetaminophen-induced liver injury is attenuated in *Trpm2* KO mice compared to WT animals.

#### 4.3.2. Radiation-Induced Tissue Damage

Radiation is a mainstay of treatment for head and neck cancer. Unfortunately, it has significant adverse effects on healthy tissues that are in the field of the treatment. For example, xerostomia (dry mouth) is a result of salivary gland damage by radiation. The molecular mechanism of radiation injury is complex including generation of ROS and DNA damage [110]. TRPM2 was reported to exacerbate radiation-induced salivary gland dysfunction [110]. H_2_O_2_ and radiation induced the influx of Ca^2+^ in salivary gland acinar cells. This influx was reduced by the disruption of *Trpm2*. The irreversible loss of salivary gland fluid secretion in WT mice subjected to radiation was improved by using free radical scavengers and/or PARP inhibitors.

#### 4.3.3. Alzheimer’s Disease

Alzheimer’s disease (AD) is a devastating form of progressive dementia of unknown cause and no effective treatment. Therefore, it is an attractive hypothesis that TRPM2 may be involved in neuronal cell death in AD patients [111]. The suggested pathology of AD includes an alteration in the proteolytic processing of the amyloid precursor protein, APP. In addition, amyloid β-peptide (Aβ) is accumulated in the AD brain. Although the molecular defect responsible for AD remain unknown, dysregulation of Ca^2+^ homeostasis is widely believed to be intimately associated with Aβ toxicity [112]. Proposed mechanisms of Aβ neurotoxicity include the production of ROS, as well as excitotoxicity with the intracellular accumulation of Ca^2+^ [113].

Previously, Lustbader *et al* reported that Aβ-binding alcohol dehydrogenase directly interacted with Aβ in the mitochondria of both AD patients and transgenic mice. This interaction promotes the leakage of ROS [114]. A recent study suggested that TRPM2 is involved in Aβ-induced neurotoxicity [112]. APP/PS1 animals are double transgenic mice that express a chimeric mouse/human APP and overproduce Aβ. They are widely used as model of AD. APP/PS1 mice were crossed with *Trpm2* KO animals. Synapse loss and decreased levels of synaptic proteins are early correlates of the severity of AD [112]. The level of the synaptic marker, synaptophysin, in the hippocampus was found to be lower in APP/PS1 mice than in WT mice. Synaptophysin levels in the hippocampus of *Trpm2*-disrupted APP/PS1 mice were similar to those in WT mice. In addition, age-dependent spatial memory deficits in APP/PS1 mice were reversed in *Trpm2*-disrupted APP/PS1 mice. These observations imply an important role for TRPM2 in Aβ-induced neuronal toxicity.

In addition, AD is associated with reductions in cerebral blood flow early in the course of the disease [115]. This Aβ-induced cerebrovascular dysfunction may be mediated by TRPM2 [115]. Indeed, TRPM2 is expressed in brain endothelial cells; in these cells, Aβ stimulated the influx of Ca^2+^ via TRPM2. The Aβ-induced activation of TRPM2 in brain endothelial cells was mediated by ROS derived from the activation of NADPH oxidase. Reduction in cerebral blood flow was induced by the neocortical superfusion of Aβ, and was attenuated by PARP and PARG inhibitors, as well as the disruption of *Trpm2*. Combined, these findings indicate that Aβ-induced TRPM2 activation contributes to endothelial dysfunction.

## 5. Conclusions

There is increasing evidence that TRPM2 plays an important role in the pathomechanism of ROS-coupled diseases. For example, TRPM2 contributes to aggravation of disease states in which monocytes/macrophages play a pivotal role via cytokine production. In the brain and kidney, TRPM2 is involved in ischemia-reperfusion injury. Moreover, TRPM2 is implicated in innate immunity and the pathobiology of Alzheimer disease.

**Figure 2:**
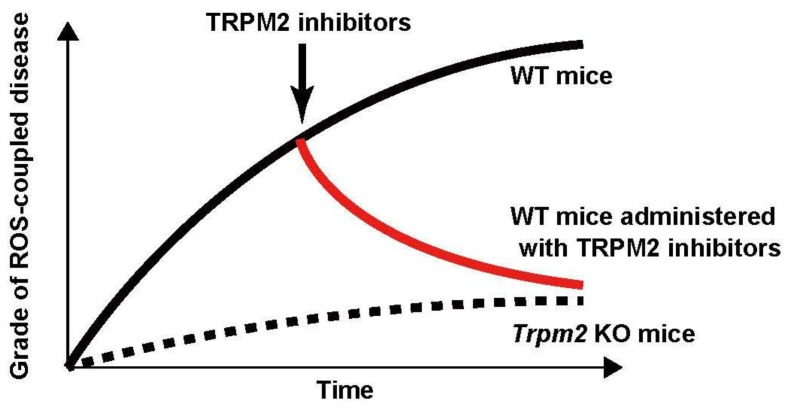
Does administration with TRPM2 inhibitors during ROS-coupled disease development improve the grade of these diseases? Pathological mouse model studies have been performed under *Trpm2*-disrupted conditions, and suggested that *Trpm2* KO mice are protected from ROS-coupled diseases. However, in terms of cure, it is important that the grade of these diseases is improved by the inhibition of TRPM2 during disease development. Therefore, the studies whether the inhibition of TRPM2 during ROS-coupled disease development has curative effects on the diseases should be done in the future.

On the other hand, there are conflicting findings with regard to the function of TRPM2 in myocardial infarction and in LPS-induced lung injury. Non-specific TRPM2 inhibitors (e.g., PARP inhibitors, clotrimazole, and 2-APB) have been shown to attenuate the exacerbation of ROS-coupled diseases. For instance, PARP inhibitors were found to attenuate radiation-induced tissue damage [110], and bleomycin-induced lung injury [85]. Furthermore, clotrimazole attenuated heart [105] and brain damage [98,100] induced by ischemia-reperfusion. 2-APB also exerted protective effects against ischemia-reperfusion-induced brain [102] and kidney [106] damage (Figure 2). These promising results, however, must be confirmed by yet-to-be-synthesized selective TRPM2 antagonists.
